# Artificial Intelligence-Driven Fractional Flow Reserve Assessment: Technical Foundations, Clinical Insights, and Future Directions

**DOI:** 10.3390/medicina62061157

**Published:** 2026-06-14

**Authors:** Abdelrahman Hafez, Kamal Awad, Juan M. Farina, Mohamed Nour, Mohamed Reyad Mohamed, Isabel G. Scalia, Sherif Ahmed, Fatmaelzahraa Abdelfattah, Mahshad Razaghi, Laurève Chollet, Cecilia Villa Etchegoyen, Ramzi Ibrahim, Balaji Tamarappoo, Matthew Stib, Chadi Ayoub, Reza Arsanjani

**Affiliations:** 1 Department of Cardiovascular Medicine, Mayo Clinic, Phoenix, AZ 85054, USA; awad.kamal@mayo.edu (K.A.); farina.juanmaria@mayo.edu (J.M.F.); nour.mohamed@mayo.edu (M.N.); scalia.isabel@mayo.edu (I.G.S.); ahmed.sherif@mayo.edu (S.A.); abdelfattah.fatmaelzahraa@mayo.edu (F.A.); razaghi.mahshad@mayo.edu (M.R.); chollet.laureve@mayo.edu (L.C.); villaetchegoyen.cecilia@mayo.edu (C.V.E.); ibrahim.ramzi@mayo.edu (R.I.); tamarappoo.balaji@mayo.edu (B.T.); ayoub.chadi@mayo.edu (C.A.); arsanjani.reza@mayo.edu (R.A.); 2 Department of Internal Medicine, College of Medicine, University of Arizona, Phoenix, AZ 85004, USA; mohamed.mohamed@bannerhealth.com; 3 Department of Radiology, Mayo Clinic, Phoenix, AZ 85259, USA; stib.matthew@mayo.edu

**Keywords:** fractional flow reserve, artificial intelligence, deep learning, non-invasive imaging, functional assessment, coronary artery disease

## Abstract

Coronary artery disease (CAD) remains a leading cause of global morbidity and mortality. Accurate diagnosis of ischemia-causing coronary stenoses is essential for guiding revascularization and improving outcomes. Although invasive fractional flow reserve (FFR) remains the gold standard for functional lesion assessment, its use is limited by procedural invasiveness, cost, and complexity. CT-derived FFR (FFRct), based on computational fluid dynamics (CFD), was the first major advance in noninvasive physiological assessment, but its adoption has been hindered by intensive off-site computation and dependence on high-quality imaging. This review summarizes the evolution from invasive FFR to AI-driven functional assessment of coronary lesions. We examine the principles and validation of CFD-based FFRct and then focus on the shift toward artificial intelligence, including both machine learning (ML) and deep learning (DL) approaches. These methods range from models using engineered geometric and plaque features trained on large synthetic datasets to end-to-end systems that learn directly from imaging data. We discuss key validation studies evaluating diagnostic accuracy, prognostic value, and clinical utility, with attention to performance in challenging settings such as intermediate stenoses, heavy calcification, and patients with comorbidities. We also highlight major barriers to widespread adoption, including dependence on input data quality, limited explainability, regulatory hurdles, and integration into clinical workflows. Finally, we outline future directions, including AI-enabled virtual PCI planning, multimodal risk stratification, and broader access to functional cardiac assessment. AI has the potential to transform noninvasive coronary imaging by enabling a single CCTA scan to provide rapid, integrated evaluation of anatomy, plaque characteristics, and physiological significance, supporting more personalized care and better clinical outcomes.

## 1. Introduction

Coronary artery disease (CAD) represents a global health crisis, standing as the foremost cause of mortality worldwide [1]. The pathophysiology of CAD involves the progressive buildup of atherosclerotic plaque within the walls of the coronary arteries, which can lead to luminal narrowing, impede myocardial blood flow, and precipitate a spectrum of clinical events from stable angina to acute myocardial infarction (MI) and sudden cardiac death [2]. For decades, the cornerstone of diagnosis and treatment planning has been invasive coronary angiography (ICA), a technique that provides a two-dimensional silhouette of the coronary lumen, allowing for the visual estimation of stenosis severity.

However, clinical evidence has exposed a fundamental limitation of a purely anatomical approach: the degree of luminal narrowing on angiography correlates imperfectly with the physiological significance of a coronary lesion [3]. Contemporary literature has further emphasized that anatomical disease burden alone does not fully define the expected benefit of an invasive strategy, reinforcing the importance of integrating physiological, anatomical, and clinical risk information when selecting patients for revascularization [4]. This well-documented anatomy–physiology discordance led to the “oculostenotic reflex,” a tendency to revascularize lesions based on their visual severity alone. This practice resulted in a significant number of inappropriate interventions on lesions that were not causing ischemia, while potentially leaving physiologically significant lesions untreated [5].

The paradigm shifted with the advent of invasive fractional flow reserve (FFR). FFR is a pressure-wire-based index measured during maximal coronary hyperemia (typically induced by adenosine) that quantifies the physiological impact of a stenosis. It is defined as the ratio of the pressure distal to a stenosis (Pd) to the pressure proximal to it in the aorta (Pa). An FFR value of ≤0.80 has been robustly validated as the threshold for identifying ischemia-producing lesions [6]. In parallel, non-hyperemic pressure ratios, particularly the instantaneous wave-free ratio (iFR), have emerged as adenosine-free invasive alternatives to FFR. In DEFINE-FLAIR and iFR-SWEDEHEART, iFR-guided revascularization was noninferior to FFR-guided revascularization with respect to major adverse cardiovascular events, supporting a broader physiology-guided paradigm beyond adenosine-dependent FFR alone [7,8]. The seminal FAME (Fractional Flow Reserve versus Angiography for Multivessel Evaluation) and FAME 2 trials irrefutably established the clinical superiority of an FFR-guided revascularization strategy. These trials demonstrated that guiding PCI with FFR, rather than angiography alone, led to significantly better clinical outcomes, including lower rates of death, MI, and repeat revascularization, all while reducing the number of stents used and lowering healthcare costs [9,10].

Despite this high-level evidence, invasive FFR remains underused in routine practice, with adoption varying substantially across regions, institutions, and clinical scenarios [11]. The barriers are practical and substantial: the procedure is invasive, requires specialized equipment, adds time and cost to the catheterization, and the requisite administration of adenosine can cause significant patient discomfort, including chest pain, dyspnea, and transient atrioventricular block [11,12].

In addition to pressure-wire and non-hyperemic invasive indices, angiography-derived wire-free physiological indices have gained increasing attention. Quantitative flow ratio (QFR), vessel fractional flow reserve, FFRangio, contrast-flow quantitative flow ratio, and Murray-law-based μQFR estimate lesion physiology from invasive angiographic images without the need for a pressure wire or assessment of pharmacologic hyperemia. Although these techniques fall outside the primary CT-centered focus of this review, they are important to acknowledge because they illustrate the broader shift toward image-derived, wire-free assessment of coronary physiology [13,14,15].

Together, the limitations of conventional invasive FFR and the emergence of image-based physiological assessment highlight a compelling unmet clinical need for a non-invasive, accurate, efficient, and widely accessible approach to determine the functional significance of coronary stenoses. Recent critical appraisals have also emphasized the evolving evidence base, implementation barriers, and remaining limitations of CT-derived FFR in contemporary practice [16]. Accordingly, this review explores the current landscape of non-invasive coronary flow evaluation technologies, appraises their diagnostic performance and clinical utility, and examines their potential to overcome key limitations of invasive FFR.

### Review Methodology and Scope

**Figure 1:**
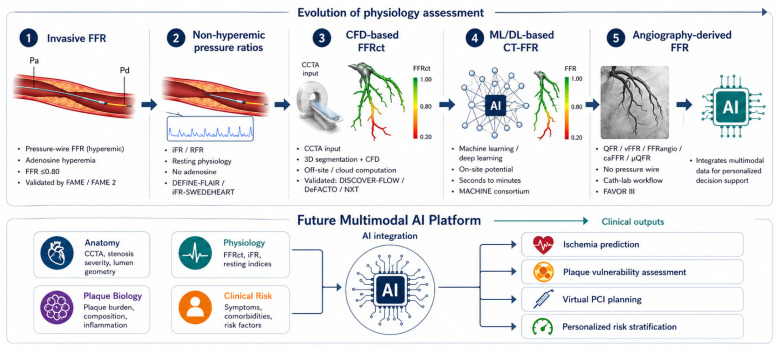
Evolution of coronary physiological assessment from invasive pressure-wire FFR to AI-enabled multimodal platforms. The upper panel summarizes the progression from invasive hyperemic FFR to non-hyperemic pressure ratios, CFD-based FFRct, ML/DL-based CT-FFR, and angiography-derived wire-free physiological indices. The lower panel illustrates a future multimodal AI platform integrating anatomy, physiology, plaque biology, and clinical risk to support ischemia prediction, plaque vulnerability assessment, virtual PCI planning, and personalized risk stratification. Abbreviations: AI, artificial intelligence; CCTA, coronary computed tomography angiography; CFD, computational fluid dynamics; CT-FFR, CT-derived fractional flow reserve estimated using machine learning/deep learning methods; DL, deep learning; FFR, fractional flow reserve; FFRct, CT-derived fractional flow reserve; iFR, instantaneous wave-free ratio; ML, machine learning; PCI, percutaneous coronary intervention; QFR, quantitative flow ratio; RFR, resting full-cycle ratio; vFFR, vessel fractional flow reserve.

This article is structured as a narrative review. We performed a targeted literature search of PubMed/MEDLINE, Embase, and major cardiovascular imaging and interventional cardiology literature using combinations of the following terms: “fractional flow reserve,” “FFRct,” “CT-derived fractional flow reserve,” “artificial intelligence,” “machine learning,” “deep learning,” “coronary computed tomography angiography,” “radiomics,” “plaque analysis,” “quantitative flow ratio,” “angiography-derived FFR,” and “software as a medical device.” Priority was given to pivotal validation studies, multicenter registries, randomized trials, guideline documents, regulatory sources, and recent reviews. The aim was to provide a clinically oriented synthesis of the technical evolution, validation evidence, implementation barriers, and future directions of AI-enabled coronary physiological assessment (Figure 1).

## 2. The First Non-Invasive Frontier: FFR from Computed Tomography (FFRct)

The first major breakthrough in answering this need came from the innovative application of advanced physics to medical imaging. CT-derived FFR (FFRct) (FFRct; HeartFlow FFRCT Analysis, HeartFlow, Inc., Redwood City, CA, USA) pioneered a method to calculate FFR non-invasively using data from a standard, high-quality coronary computed tomography angiography (CCTA) scan.

### 2.1. Principles of CFD-Based FFRct

The technology is founded on the principles of computational fluid dynamics (CFD). A patient-specific 3D anatomical model of the coronary tree is meticulously segmented from the CCTA data. Using this model, boundary conditions are established based on physiological principles, such as allometric scaling laws, to model the resistance of the downstream microvasculature. Powerful off-site supercomputers then solve the complex Navier–Stokes equations of fluid motion, simulating blood flow and pressure under conditions of maximal hyperemia. The result is a color-coded 3D model of the patient’s coronary arteries displaying FFR values at every point, allowing for a comprehensive physiological map without any additional imaging, radiation, or medication [17,18].

### 2.2. Validation and Clinical Utility

The diagnostic performance of FFRct was rigorously validated in a series of prospective, multicenter diagnostic performance trials using invasive FFR measured during coronary angiography as the reference standard. In the first-in-human DISCOVER-FLOW study, patients underwent CCTA followed by invasive coronary angiography with pressure-wire FFR, and FFRct was computed from standard CCTA datasets to test whether it could correctly identify lesion-specific ischemia (FFR ≤ 0.80); this provided the initial proof of concept that physiologic significance could be derived noninvasively from anatomy plus modeling. The subsequent DeFACTO trial extended this validation in a larger, international multicenter cohort with blinded core-laboratory interpretation of CT, invasive angiography, and FFR/FFRct, comparing FFRct against invasive FFR for diagnosing hemodynamically significant CAD [19,20]. The pivotal NXT (Analysis of Coronary Blood Flow Using Coronary CT Angiography: NeXT sTeps) trial compared FFRct to invasive FFR in 254 patients, demonstrating a high per-patient diagnostic accuracy of 81% and an area under the curve (AUC) of 0.90, which was significantly superior to the performance of CCTA anatomical assessment alone (AUC 0.81) [21]. Nevertheless, these early trials should be interpreted as proof of concept and early-generation algorithm evaluations rather than fixed estimates of contemporary performance. Across validation studies, diagnostic accuracy was generally good but not perfect, and performance was lowest near the clinical decision threshold around 0.80. Cook et al. demonstrated that diagnostic accuracy varies across the FFRct spectrum and is particularly limited in the clinically relevant gray zone [22]. In addition, a clinically meaningful proportion of CCTA datasets may be non-evaluable for FFRct because of motion artifact, severe calcification, poor contrast opacification, or segmentation limitations [23,24].

Beyond diagnostic accuracy, the PLATFORM (Prospective Longitudinal Trial of FFRCT: Outcome and Resource Impacts) study demonstrated clinical utility. In a cohort of patients with stable angina, an FFRct-guided strategy led to a reduction in the rate of invasive angiography that revealed no obstructive disease (from 73% in the usual care arm to just 12% in the FFRct arm). This confirmed FFRct’s role as an effective gatekeeper to the cath lab [25]. Furthermore, long-term follow-up studies, such as the PROMISE trial sub-study and the ADVANCE registry, have shown that an abnormal FFRct value (≤0.80) is a stronger independent predictor of future major adverse cardiovascular events (MACE) than the presence of severe anatomical stenosis on CCTA [26,27]. Real-world data from the ADVANCE registry further supported the clinical utility of FFRct by showing that FFRct influenced downstream management and that patients with negative FFRct values had low rates of cardiovascular death or myocardial infarction during follow-up [27,28]. This robust body of evidence led to the inclusion of FFRct in major societal guidelines in the United States and Europe as a Class 2a recommendation for evaluating intermediate coronary stenoses [29]. Consistent with this literature, the 2021 AHA/ACC/ASE/CHEST/SAEM/SCCT/SCMR Chest Pain Guideline supports the use of FFRct as an adjunct to CCTA in selected patients with intermediate coronary stenoses to improve diagnostic specificity and guide downstream testing or invasive angiography [29].

It is also important to recognize that invasive FFR ≤ 0.80, although clinically validated, is not an infallible reference standard. FFR measurements may be affected by pressure-wire position, pressure drift, serial stenoses, microvascular function, and adequacy of pharmacologic hyperemia. Moreover, the emergence of iFR and other non-hyperemic pressure ratios has broadened the invasive physiology landscape. Therefore, the diagnostic performance of FFRct and AI-derived FFR should be interpreted against the biological and technical variability of invasive physiology itself, particularly near the 0.80 decision threshold [8,22] (Table 1).

### 2.3. Limitations of the CFD-Based Approach

While transformative, the CFD-based FFRct model is not without limitations that have hindered its universal adoption. The primary constraint is its reliance on off-site, high-performance computing, which results in a turnaround time of several hours, rendering it unsuitable for acute or emergency settings. The associated costs and complex reimbursement landscape have also posed barriers. Most critically, the accuracy of the entire simulation is contingent upon the quality of the source CCTA images. Image artifacts caused by motion, severe calcification (blooming artifacts), or suboptimal contrast opacification can make the scan “unevaluable” for FFRct analysis, a scenario that occurred in up to 25% of patients in some early studies [23,24,30]. These challenges paved the way for the next technological evolution: the application of artificial intelligence (AI).

## 3. The Paradigm Shift: Artificial Intelligence in FFR Estimation

Artificial intelligence, particularly its subfields of machine learning (ML) and deep learning (DL), offers a fundamentally different approach to problem-solving. Rather than relying on first principles of physics, AI algorithms learn to recognize complex patterns and make predictions by training on vast amounts of data [31]. This has opened several new, promising avenues for non-invasive FFR estimation that address the limitations of the CFD approach.

### 3.1. Machine Learning Approaches (CT-FFR_ML)

The first wave of AI-FFR models employed “classical” ML algorithms. This approach involves a two-step process. First, human experts or sophisticated software extract a set of predefined geometric and anatomical features from the segmented coronary arteries. These features can include lesion length, minimum lumen diameter, percent area stenosis, plaque volume and composition, vessel tortuosity, and branching angles. In the second step, these curated features are fed into an ML algorithm (such as a support vector machine, random forest, or gradient boosting model), which is trained to learn the complex, non-linear relationship between these anatomical inputs and the invasively measured FFR output [32].

A key methodological innovation in this area, detailed by Tesche et al., was the training of an ML model on a massive synthetic database. Researchers algorithmically generated a database of 12,000 diverse 3D coronary artery models, encompassing a wide range of stenosis severities, locations, and vessel geometries. For each synthetic model, a CFD simulation was run to generate the “ground truth” FFR value. The ML algorithm was then trained on this dataset to learn the manifold relationship between anatomy and its hemodynamic consequences [33,34]. This synthetic-data strategy was a key methodological innovation because it exposed the algorithm to a broader range of stenosis severities, vessel sizes, branching patterns, and lesion geometries than would have been feasible using patient data alone. Itu et al. demonstrated that such an ML approach could rapidly compute CT-derived FFR from coronary anatomy, while the MACHINE consortium subsequently validated an on-site ML-based CT-FFR approach in a multicenter cohort and showed improved diagnostic performance compared with anatomical CCTA stenosis assessment alone [34,35].

The clinical validation of this ML approach (CT-FFR_ML) was demonstrated in the retrospective, multicenter MACHINE registry. In this study of 351 patients, CT-FFR_ML significantly improved diagnostic accuracy for detecting hemodynamically significant CAD compared to CCTA stenosis assessment alone (78% vs. 58%). Crucially, its performance was not significantly different from the computationally intensive off-site CFD-based FFRct (AUC 0.84 for both), but the analysis could be performed on-site in minutes [35].

Further sub-studies from the MACHINE registry have demonstrated the robustness of the CT-FFR_ML algorithm across various challenging clinical scenarios:

- Intermediate Stenosis: CT-FFR_ML showed significantly improved performance in identifying ischemia-causing lesions in the challenging 30–69% stenosis category compared to CCTA alone (AUC 0.79 vs. 0.53) [36].
- Coronary calcification: Severe coronary calcification remains a major challenge for CCTA because blooming artifacts may obscure the true lumen boundary. In the MACHINE registry, Tesche et al. showed that ML-based CT-FFR maintained superior diagnostic performance compared with CCTA alone across coronary calcium categories, although performance was affected in patients with high calcium burden [37].
- Comorbidities: In patients with diabetes mellitus, CT-derived FFR appears to maintain diagnostic performance, although interpretation may be complicated by diffuse atherosclerosis and microvascular dysfunction. Eftekhari et al. showed that the diagnostic performance of FFRct was independent of hypertension and diabetes, and the MACHINE consortium similarly reported comparable diagnostic performance of ML-based CT-FFR in patients with versus without diabetes [38,39].
- Sex-Specific Performance: The algorithm demonstrated a statistically significant improvement in diagnostic accuracy in men (258 patients, 398 vessels). In contrast, this superiority was not observed in the smaller cohort of women (93 patients, 127 vessels), likely due to limited statistical power [40].

### 3.2. Deep Learning Approaches

Deep learning represents a more advanced subset of artificial intelligence. Deep learning models, particularly convolutional neural networks, use multilayer architectures to learn hierarchical image features directly from imaging data, thereby reducing dependence on predefined human-engineered descriptors [41].

In the context of FFR estimation, a deep learning system may be trained on CCTA datasets paired with invasive FFR values to learn image-derived patterns related to vessel geometry, plaque burden, and downstream physiological significance. Kumamaru et al. developed a fully automated 3D deep learning model for estimating patient-level minimum FFR from CCTA, achieving an AUC of 0.78 and an accuracy of 76% for detecting abnormal FFR [42].

The application of deep learning is not limited to CCTA. Koifman et al. described an angiography-based artificial intelligence approach for real-time, wire-free, adenosine-free FFR estimation in the catheterization laboratory; the reported pilot performance included 88% sensitivity, 93% specificity, and 90% overall accuracy compared with invasive FFR [12].

### 3.3. Integrating Plaque and Radiomics

The latest evolution in AI-driven FFR assessment is the movement beyond luminal geometry alone toward integration of plaque biology, perivascular tissue information, and automated image interpretation. CCTA-derived plaque features, including low-attenuation noncalcified plaque, positive remodeling, the napkin-ring sign, and total plaque burden, provide information related to plaque vulnerability and future cardiovascular risk [43,44].

Radiomics refers to the high-throughput extraction of quantitative image features from medical images, capturing intensity, shape, texture, and heterogeneity patterns that may not be visually apparent [45]. In coronary imaging, radiomic plaque features have been shown to outperform conventional quantitative CT metrics for identifying high-risk plaque phenotypes such as the napkin-ring sign [46]. Broader cardiac-imaging AI reviews and contemporary deep-learning CCTA studies further support the integration of automated plaque and stenosis quantification into risk prediction workflows [47,48]. In parallel, machine learning CCTA studies have shown that quantitative plaque analysis can improve ischemia prediction beyond visual stenosis assessment alone [32]. Therefore, future AI-FFR platforms will likely combine luminal anatomy, plaque composition, radiomic signatures, and physiological estimates to provide a more comprehensive assessment of lesion significance and patient-level risk (Table 2 and Table 3).

## 4. Challenges to Clinical Adoption

Despite the immense promise and rapid technological progress, several significant hurdles must be overcome for the widespread and responsible adoption of AI-FFR into routine clinical practice.

### 4.1. Data Quality Dependency

The foundational principle of “garbage in, garbage out” remains paramount. The performance of any non-invasive FFR method, whether CFD- or AI-based, is critically dependent on the quality of the source CCTA scan. Severe calcification, which causes blooming artifacts that obscure the true lumen boundary, remains a formidable challenge. Motion artifacts from high heart rates or poor breath-holds, and stair-step artifacts in arrhythmia, can lead to inaccurate vessel segmentation and, consequently, erroneous FFR predictions. Adherence to standardized, guideline-recommended acquisition protocols, including the use of beta-blockers and nitroglycerin, is essential to ensure the high image quality required for these advanced analyses [49].

Because both CFD- and AI-based FFR estimation depend on accurate coronary lumen segmentation, image quality remains central to diagnostic reliability. Motion artifact, high heart rate, severe calcification, suboptimal contrast opacification, and stair-step artifact can impair segmentation and reduce diagnostic performance. Dedicated CCTA acquisition protocols, including ECG gating, heart-rate optimization, nitroglycerin administration when appropriate, and adequate contrast timing, are therefore essential for reliable FFRct or AI-FFR analysis [24,49].

### 4.2. The “Black Box” Problem and Explainability

Many high-performing DL models function as “black boxes,” producing a physiological estimate without a transparent explanation of the image regions or features driving the prediction. Explainable AI approaches may partially address this limitation. Post hoc methods such as saliency maps, class activation maps, SHAP, LIME, and attention visualization can highlight image regions or variables that influenced model output. However, post hoc explanations do not necessarily prove that the model is using clinically meaningful causal features. Intrinsically interpretable models, uncertainty quantification, and hybrid physics-informed architectures may therefore be particularly important for Software as a Medical Device applications, where clinicians and regulators require not only accuracy but also reliability, traceability, and safe handling of out-of-distribution cases [50,51,52].

### 4.3. Generalizability and Robustness

An AI model is only as good as the data it was trained on. A model developed at a single institution using data from a specific scanner and patient population may not perform well when applied to the more heterogeneous data encountered in the real world. To be clinically useful, an AI-FFR tool must be robust and generalizable across different scanners, reconstruction algorithms, patient ethnicities, and disease prevalences. This requires rigorous external validation on large, diverse, multi-center, multi-vendor datasets before widespread deployment [51].

Another important source of variability is software platform heterogeneity. CT-FFR values may differ across vendors because of differences in segmentation algorithms, boundary-condition assumptions, computational models, and post-processing workflows. Therefore, validation should be platform-specific rather than assuming interchangeability across CT-FFR software packages [53].

### 4.4. Regulatory and Reimbursement Landscape

AI-enabled tools that provide diagnostic or treatment-guiding information are regulated as Software as a Medical Device. In the United States, the FDA has finalized guidance on Predetermined Change Control Plans for AI-enabled device software functions, allowing manufacturers to prospectively define certain algorithm modifications and the methodology for validating and implementing them while maintaining reasonable assurance of safety and effectiveness [54]. In Europe, the EU AI Act entered into force on 1 August 2024 and will become applicable in stages, including specific obligations for high-risk AI systems embedded in regulated products [55,56]. These frameworks emphasize risk management, transparency, human oversight, post-market monitoring, and documentation of algorithm changes. In parallel, reimbursement remains a practical barrier; without clear payment pathways, even technically validated AI-FFR tools may face limited clinical adoption.

### 4.5. Seamless Workflow Integration

For any new technology to succeed, it must integrate smoothly into the existing clinical workflow of a busy radiology department or cardiology practice. The on-site, rapid analysis offered by AI-FFR is a major advantage over off-site CFD models. The ideal implementation would be a fully automated system, perhaps cloud-based, that integrates directly with the Picture Archiving and Communication System (PACS) and the reporting software. Such a system would automatically process eligible CCTA scans and present the functional assessment alongside the anatomical images, with minimal additional clicks or manual input required from the radiologist or cardiologist.

## 5. Future Directions

The field of AI-driven functional cardiac assessment is dynamic and advancing at an extraordinary pace. The future is likely to be shaped by several key developments that will further enhance the power and utility of these tools.

- Virtual Intervention Planning: A major emerging application is the use of AI for “virtual PCI” or treatment planning. After identifying a physiologically significant lesion using AI-FFR, the clinician could use a “CT-FFR Planner” tool to simulate the placement of a virtual stent of a specific length and diameter. The system would then predict the post-intervention FFR, allowing the physician to optimize the revascularization strategy and, importantly, to identify patients with diffuse, non-focal disease who may derive little physiological benefit from stenting a single segment [57].
- Holistic, Multi-Modal Risk Stratification: The ultimate goal is to move beyond a simple binary diagnosis of ischemia towards a comprehensive, personalized risk assessment. Future AI platforms will likely integrate multiple data streams from a single CCTA scan, including anatomical data (stenosis grade), detailed plaque analysis (volume, composition, high-risk features), and functional data (AI-FFR), to generate a single, powerful, and personalized risk score. This score could predict a patient’s long-term risk of MACE with far greater accuracy than any single parameter alone [58].

Future AI platforms will likely integrate multiple data streams from a single CCTA scan, including stenosis severity, plaque volume and composition, high-risk plaque features, perivascular tissue characteristics, and CT-derived physiological estimates. This direction is supported by emerging deep-learning CCTA studies showing that automated plaque and stenosis quantification can provide prognostic information beyond conventional visual assessment [48].

Hybrid physics-informed neural networks may represent an additional future direction by embedding hemodynamic constraints into learning architectures. Such approaches could potentially improve biological plausibility, reduce dependence on purely empirical pattern recognition, and enhance robustness in settings where labeled invasive FFR data are limited [41].

- Democratization of Functional Assessment: Although AI-FFR may broaden access by reducing computational burden and enabling rapid on-site or cloud-based analysis, democratization is not automatic. If these tools require high-end CT scanners, specialized acquisition protocols, or costly software licenses, they may widen rather than reduce disparities. Equitable implementation will require attention to cost, interoperability, reimbursement, cloud infrastructure, and validation in lower-resource settings.

## 6. Conclusions

The evolution from invasive FFR to AI-driven FFR estimation represents a remarkable and rapid convergence of clinical medicine, biomedical engineering, and computer science. While CFD-based FFRct laid the critical groundwork and proved the principle of non-invasive physiological assessment, AI is now poised to overcome its key limitations in speed, cost, and automation. By offering a rapid, robust, and increasingly sophisticated analysis that integrates anatomy, plaque biology, and physiology, AI is fundamentally transforming the role of CCTA from a simple anatomical rule-out test into a comprehensive single-study solution for the assessment of coronary artery disease. While significant challenges related to validation, explainability, regulation, and integration must continue to be addressed with scientific rigor, the relentless advancement of these technologies heralds a new era in personalized cardiology, where critical treatment decisions are guided by a more complete, non-invasive understanding of each patient’s unique coronary disease process.

## Figures and Tables

**Table 1:** Summary of Foundational Validation Trials for CFD-Based FFRct.

Trial Acronym (Reference)	Study Design	Patient Population (*n*)	Key Endpoint/Comparison	Key Findings	Clinical Implication
**NXT** [21]	Prospective, multicenter, diagnostic accuracy	Suspected CAD referred for ICA (*n* = 254)	Diagnostic accuracy of FFRct vs. invasive FFR	Per patient: 81% accuracy, 86% sensitivity, 79% specificity, and AUC 0.90. Superior to CCTA alone (AUC 0.81).	Established FFRct as a highly accurate non-invasive alternative to invasive FFR for diagnosing significant CAD.
**PLATFORM** [25]	Prospective, multicenter, comparative	Stable chest pain patients planned for ICA (*n* = 584)	rate of ICA showing no obstructive CAD	FFRct-guided strategy reduced non-obstructive ICA from 73% to 12%. 61% of planned ICAs were canceled.	Validated FFRct as an effective gatekeeper, reducing unnecessary invasive procedures and associated costs.
**PROMISE Sub-study** [26]	Retrospective analysis of prospective trial data	Symptomatic suspected CAD patients (*n* = 10,030)	Prognostic value for predicting MACE	FFRct ≤ 0.80 was a stronger predictor of 2-year MACE than severe CCTA stenosis (HR 5.01 vs. 3.75).	Reinforced FFRct’s prognostic value, adding critical risk information beyond anatomical assessment.
**PACIFIC-1** [30]	Prospective, multicenter, diagnostic accuracy	Suspected CAD referred for ICA (*n* = 208)	Diagnostic accuracy vs. invasive FFR, PET, and SPECT	FFRct showed superior diagnostic performance (AUC 0.94) vs. CCTA, SPECT, and PET for ischemia detection.	Positioned FFRct as superior to other non-invasive functional tests for CAD evaluation.

AUC: area under the receiver operating characteristic curve; CAD: coronary artery disease; CCTA: coronary computed tomography angiography; FFR: fractional flow reserve; FFRct: CT-derived FFR; HR: hazard ratio; ICA: invasive coronary angiography; MACEs: major adverse cardiovascular events; PET: positron emission tomography; SPECT: single-photon emission computed tomography.

**Table 2:** Summary of key validation studies for AI-based FFR estimation.

Study/First Author (Reference)	AI Approach/Innovation	Imaging Modality	Patient Cohort (*n*)	Key Performance Metrics (vs. Invasive FFR)	Key Conclusion
**Itu et al. ^‡^ (2016)** [34]	ML model trained on 12,000 synthetic coronary anatomies and validated in patient-specific CCTA models	CCTA	87 patients/125 lesions	Accuracy 83.2%; sensitivity 81.6%; specificity 83.9%; AUC 0.90 vs. invasive FFR	Demonstrated that synthetic-data-trained ML could provide rapid CT-derived FFR estimation with strong agreement against invasive FFR and markedly reduced computation time.
**Coenen et al. (2018)—MACHINE** [35]	On-site ML-based FFR (CT-FFR_ML)	CCTA	351 patients	Per-vessel: 78% accuracy, 81% sensitivity, 76% specificity, AUC 0.84	First large multicenter validation of an on-site ML approach; comparable to CFD-based methods.
**Kumamaru et al. (2020)** [42]	Fully automated 3D DL model	CCTA	1052 vessels	Per-vessel: 76% accuracy, 85% sensitivity, 63% specificity, AUC 0.78	Demonstrated feasibility of a fully automated, end-to-end DL pipeline for FFR with minimal user input.
**Koifman et al. (2022)** [12]	DL algorithm for real-time FFR estimation	Invasive Coronary Angiography (ICA)	31 patients	90% accuracy, 88% sensitivity, 93% specificity	Introduced wire-free, adenosine-free FFR from cath lab angiograms using DL.
**Lin et al. (2022)** [48]	ML model with quantitative plaque analysis	CCTA	581 vessels	AUC 0.92 for predicting ischemia; outperformed visual assessment (AUC 0.84)	Demonstrated advantage of integrating plaque metrics into ML for improved prediction.

AI: artificial intelligence; AUC: area under the receiver operating characteristic curve; CCTA: coronary computed tomography angiography; CFD: computational fluid dynamics; DL: deep learning; FFR: fractional flow reserve; ICA: invasive coronary angiography; ML: machine learning. ^‡^: For Itu et al., the 12,000 synthetic coronary models refer to the training dataset, whereas the 87 patients/125 lesions refer to the clinical validation cohort with invasive FFR comparison.

**Table 3:** Comparative overview of non-invasive coronary assessment technologies.

Feature	CCTA Stenosis Assessment	CFD-Based FFRct (e.g., HeartFlow)	AI-Based FFR (ML/DL)
**Underlying Principle**	Anatomical Luminal Narrowing	Physics-Based (Computational Fluid Dynamics)	Data-Driven (Pattern Recognition)
**Typical Turnaround Time**	Minutes	Hours (Requires off-site supercomputing)	Seconds to Minutes (On-site potential)
**Key Advantages**	Fast and widely available; excellent NPV for ruling out CAD	High diagnostic accuracy; guideline-endorsed; proven prognostic value	Extremely fast; fully automated potential; integrates plaque/radiomics; lower computational cost
**Key Limitations**	Poor physiological correlation; high false-positive rate	Slow turnaround time; high cost; requires pristine image quality; off-site data transfer	“Black box” explainability issue; requires large, diverse training data; generalizability concerns; less long-term outcome data
**Clinical Use Status**	Standard of Care	Clinically adopted; guideline-recommended	Emerging, primarily research use, some with regulatory clearance

AI: artificial intelligence; CAD: coronary artery disease; CCTA: coronary computed tomography angiography; CFD: computational fluid dynamics; DL: deep learning; FFR: fractional flow reserve; FFRct: CT-derived FFR; ML: machine learning; NPV: negative predictive value.

## Data Availability

No new primary data were used.

## Abbreviations

AI	Artificial Intelligence
AUC	Area Under the Curve
CAC	Coronary Artery Calcium
CAD	Coronary Artery Disease
CCTA	Coronary Computed Tomography Angiography
CFD	Computational Fluid Dynamics
CNNs	Convolutional Neural Networks
CT	Computed Tomography
CT-FFR_ML	Machine Learning-Based CT-Derived Fractional Flow Reserve
DL	Deep Learning
ESC	European Society of Cardiology
EACTS	European Association for Cardio-Thoracic Surgery
FAME	Fractional Flow Reserve versus Angiography for Multivessel Evaluation
FFR	Fractional Flow Reserve
FFRct	CT-Derived Fractional Flow Reserve
HR	Hazard Ratio
ICA	Invasive Coronary Angiography
MACE	Major Adverse Cardiovascular Events
MI	Myocardial Infarction
ML	Machine Learning
NASCI	North American Society for Cardiovascular Imaging
NPV	Negative Predictive Value
PCI	Percutaneous Coronary Intervention
Pd	Distal Coronary Pressure
PET	Positron Emission Tomography
PROMISE	Prospective Multicenter Imaging Study for Evaluation of Chest Pain
SPECT	Single-Photon Emission Computed Tomography
SCCT	Society of Cardiovascular Computed Tomography
